# Human Sapovirus in Clams, Japan

**DOI:** 10.3201/eid1304.061390

**Published:** 2007-04

**Authors:** Grant S. Hansman, Tomoichiro Oka, Reiko Okamoto, Tomoko Nishida, Shoichi Toda, Mamoru Noda, Daisuke Sano, You Ueki, Takahiro Imai, Tatsuo Omura, Osamu Nishio, Hirokazu Kimura, Naokazu Takeda

**Affiliations:** * National Institute of Infectious Diseases, Tokyo, Japan; † Yamaguchi Prefectural Research Institute of Public Health, Yamaguchi, Japan; ‡ Hiroshima City Institute of Public Health, Hiroshima, Japan; § Tohoku University, Sendai, Japan; ¶ Miyagi Prefectural Institute of Public Health and Environment, Sendai, Japan

**Keywords:** sapovirus, shellfish, RT-PCR, clams, Japan, dispatch

## Abstract

Human sapovirus was detected in 4 of 57 clam packages by reverse transcription–PCR and sequence analysis. This represents the first finding of sapovirus contamination in food. Closely matching sequences have been detected in stool specimens from patients with gastroenteritis in Japan, which indicates a possible food-to-human transmission link.

Sapoviruses and noroviruses are etiologic agents of human gastroenteritis. Human noroviruses are the most important cause of outbreaks of gastroenteritis worldwide and can be transmitted by a variety of routes, including food (*1*). Sapovirus infections are mostly associated with sporadic gastroenteritis in young children; however, foodborne transmission routes are yet to be determined. The most widely used method of detection is reverse transcription–PCR (RT-PCR), which has a high sensitivity and can also be used for genetic analysis. Sapovirus strains can be divided into 5 genogroups; GI–GV infect humans; sapovirus GIII infects porcine species. Phylogenetic studies have also designated sapovirus clusters or genotypes to further describe strains.

## The Study

The purpose of this study was to detect sapovirus in the clam *Corbicula japonica* (bivalve mollusk) and describe the genetic diversity of the strains. A total of 57 clam packages (30–60 clams per package) were collected from supermarkets or fish markets from 6 different areas in western Japan from December 8, 2005, to September 6, 2006. The samples were shucked, and the digestive diverticulum was removed by dissection and weighed. One gram of digestive diverticulum (10–15 clam/package) was homogenized with an Omini-mixer (Sorvall Inc., Newtown, CT, USA) in 10 mL phosphate-buffered saline. After centrifugation at 10,000× *g* for 30 min at 4°C, the supernatant was centrifuged at 100,000× *g* for 2 h (SW41 Rotor, Beckman Instruments, Inc., Fullerton, CA, USA). The pellet was resuspended in 140 μL distilled water and stored at –80°C until use.

RNA extraction and nested RT-PCR were performed as described (*2*). Briefly, for the first PCR, F13, F14, R13, and R14 primers were used; for the nested PCR, F22 and R2 primers were used. All RT-PCR products were analyzed by 2% agarose gel electrophoresis and visualized by ethidium bromide staining. RT-PCR products were excised from the gel and purified by the QIAquick gel extraction kit (QIAGEN, Hilden, Germany). Nucleotide sequences were prepared with the terminator cycle sequence kit (version 3.1, Applied Biosystems, Warrington, England) and determined with the ABI 3130 Avant sequencer (ABI, Boston, MA, USA). Nucleotide sequences were aligned with ClustalX, and the distances were calculated by Kimura’s 2-parameter method, as described elsewhere (*2*). Nucleotide sequence data determined in this study have been deposited in GenBank under accession nos. EF104251–EF104254.

Four (7%) of 57 clam packages were contaminated with sapovirus (termed Shijimi1, Shijimi2, Shijimi3, and Shijimi4). Genetic analysis of the partial capsid gene showed that these 4 sequences shared >98% nucleotide similarity and >97% amino acid identity. Phylogenetic analysis grouped these 4 sequences in the same genotype, i.e., GI/1 (Figure). Similar sequences were found on the database (Figure). Strains from this cluster likely represent the dominant genotype worldwide (*3*). Three of 4 sapovirus-positive clam packages were collected from different areas and at different times (Figure). The clam packages that were contaminated with Shijimi1 and Shijimi3 were collected from the same area, but 6 weeks apart, which indicates an ongoing sapovirus contamination or resistance in the natural environment. The seasonality of sapovirus infection in Japan is unknown; however, as with norovirus, sapovirus infections may also peak during winter, although further epidemiologic and environmental studies are needed.

**Figure:**
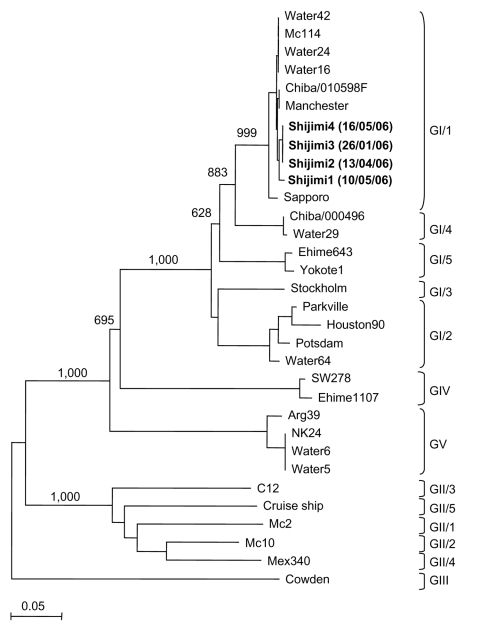
Phylogenetic analysis of sapovirus capsid sequences (≈300 nt) showing the different genogroups and clusters. Numbers on each branch indicate bootstrap values for the genotype. Bootstrap values of ≥950 were considered statistically significant for the grouping. The scale represents nucleotide substitutions per site. GenBank accession nos. for the reference strains are as follows: Arg39, AY289803; C12, AY603425; Chiba/010598F, AJ412825; Chiba000496F, AJ412800; Cruise ship, AY289804; Ehime643, DQ366345; Ehime1107, DQ058829; Houston27, U95644; Manchester, X86560; Mc2, AY237419; Mc10, AY237420; Mex340, AF435812; Parkville, U73124; Cowden, AF182760; Potsdam, AF294739; Sapporo, U65427; Stockholm, AF194182; SW278, DQ125333; water samples, DQ915088–DQ915094; and Yokote, AB253740. Boldface represents sequences detected in this study.

In a recent study, we detected sapovirus strains in 7 of 69 water samples, which included untreated wastewater, treated wastewater, and a river in Japan (*4*). Three of 7 sapovirus sequences detected in the water samples belonged to GI/1 and shared >97% nucleotide similarity with the sapovirus sequences detected in the clam packages. Additionally, sapovirus sequences belonging to GI/1 and sharing >99% nucleotide similarity, for example, Chiba/010598F strain (Figure), have been detected in stool specimens from children with sporadic gastroenteritis in Japan (*5*,*6*). The closely matching sapovirus sequences detected in the water, clams, and patients suggest that sapovirus contamination in the natural environment can lead to foodborne infections in humans, although direct evidence is lacking. More important, a recent study found animal sapovirus in oysters and suggested that coinfection with human and animal sapovirus strains could result in genomic recombination and the emergence of new strains (*7*). At the same time, we recently described the first human sapovirus intergenogroup recombinant strain (*8*). Phylogenetic analysis of the nonstructural region (i.e., genome start to capsid start) grouped this sapovirus strain in GII, while the structural region (i.e., capsid start to genome end) grouped this strain in GIV.

A large number of studies have detected norovirus in oysters. In 2 recent studies, norovirus was detected in oysters (*Crassosterea gigas*) harvested from geographically isolated areas in Japan (*9*,*10*). We also screened the same oyster samples for sapovirus; however, all of the samples were negative for sapovirus. That sapovirus was detected in the clam samples, but not in the oyster samples, is of interest. In the past several years, increasing evidence has emerged that human noroviruses bind to histo-blood group antigens (HBGAs) (*11*). These carbohydrate epitopes are present in mucosal secretions and throughout many tissues of the human body, including the small intestine, and in oyster digestive tissues. A number of studies have found that different norovirus strains exhibit different binding patterns to HBGAs and oyster digestive tissues (*12*,*13*). In a recent study, we found that sapovirus GI and GV strains showed no such binding activity to HBGAs (*14*). These results suggest that human norovirus and sapovirus strains have different binding receptors or that human sapovirus may not concentrate in detectable levels in oysters.

## Conclusions

Foodborne diseases are a major problem worldwide. We report what is, to the best of our knowledge, the first account of sapovirus contamination in food destined for human consumption. The report may represent a possible food-to-human transmission link, although direct evidence is lacking. In Japan, clams are usually boiled before they are consumed in soups. However, boiling to open the clam may not inactivate the virus (*15*); in addition, some areas in Japan do not boil clams before eating them. Further studies are needed to determine if boiling inactivates sapovirus and if the contaminated clams are indeed infectious. In conclusion, these novel results highlight the importance of sapovirus, in particular the GI/1 strains. A new awareness of sapovirus transmission routes is necessary and may help reduce sapovirus infections.
